# M. Koecker on Diseases of the Jaws

**Published:** 1828-04-01

**Authors:** 


*An Essay on Diseases of the Jaws, and their Treatment; with Observations on the Amputation of a Part or Whole of the Inferior Maxilla, &c.* By Leonard Koecker, Surgeon-Dentist, Doctor in Medicine and Surgery, &c. &c. 8vo, pp. 95. London, 1828.

The author of this little Essay is a very ingenious and scientific dentist—a regular graduate of medicine and surgery, and therefore more acquainted with medical science generally than most of the professors of dental surgery. That this extended acquaintance with the structure, functions, laws, and diseases of the human frame has not tended to make him the less a good dentist, we have the very best means of knowing—and a long and successful practice of this branch of his art in America, where diseases of the teeth and neighbouring parts are still more prevalent than here, has afforded him the most ample means of studying all the minutiæ of dental surgery, with the greatest advantage. We can confidently assure our readers, that both the author and his book are worthy of professional patronage, and we hope that patronage will not be withheld because he is a foreigner. As this Essay lies in a small compass, and embraces many interesting points of general as well as special surgery, we recommend it in the original to our readers, and shall only offer a very short analysis of its contents in this place.

In some preliminary remarks, Mr. K. has criticised, with great candour and amenity, certain opinions of Hunter and Fox, respecting diseases of the antrum and maxillary bones. Thus Mr. Hunter, in treating of diseases of the antrum inclines to the opinion, that they originate from an obliteration of the duct leading to the nose; whereas the closure of this duct is the consequence, and not the cause of the antral inflammation.

"His proposed plan of perforating the partition between the antrum and the nose, as well as of opening the inside of the lip, is not only entirely useless in a curative view, but likely to increase the disease; and very probably such treatment would never have been successful, even in the first stage of the disease, had it not been combined with better remedies, which, however, from some unhappy prejudice, or erroneous principle, were considered as secondary means, and seldom adopted until the patient had previously been subjected to painful and unnecessary operations."

Mr. Fox follows the same opinion, and observes that,

"Inflammation in the antrum is often occasioned by diseases of the teeth, but it also occurs when the teeth are quite sound. Sometimes in examining the prepared bones of the head, one or more fangs of the large molares may be found passing into the cavity. In such a case, inflammation excited by a diseased tooth is speedily communicated to the membrane lining the cavity and causes suppuration."

These views Mr. K. avers to be completely erroneous.

"The fangs of the large grinders, or, indeed, of any other tooth, never enter into the cavity of the jaw in the living subject, so long as they are possessed of vitality. Such appearances observable in anatomical preparations result from the bony structure surrounding the points of these fangs having been destroyed by the boiling or maceration in acids, or other processes, to which the maxillæ had been subjected in order to clean them from their soft parts.

"Whenever the fangs have passed into the cavity of the antrum, previously to death, they will always, together with their respective bodies, be found to have lost their vitality, the connection between them and the dental artery and nerve, the means of supporting that vitality, having been previously lost; in this state the irritation of the dead fangs produces an absorption of the osseous structure of the jaw immediately surrounding them; and occasionally inflammation and suppuration take place in what may be regarded as comparatively an early period of the disease."

Mr. K. declares it as his firm belief, the result of long experience, that diseases of both the upper and lower jaw are almost always brought on by some previous disease—disorder of the teeth, or of the parts immediately related to them.

Diseases of the maxillary bones, though not perhaps so common in this country as on the Continent, and in America, are yet by no means rare. Mr. K. has met with many instances during his residence in this metropolis, and refers to two cases successfully treated by him, that were recommended to his care by Mr. Lawrence. After enumerating the symptoms of diseases of the jaws, Mr. K. offers the following etiology.

"The proximate causes of these diseases are, as far as my experience has enabled me to judge, inflammation, suppuration, and mortification, commencing in the alveoli and the periosteum, and thence extended and communicated to the osseous structure, and the lining membrane of the cavity of the jaw.

"The exciting causes are, not only those already stated as the proximate causes of the disease, but also all diseases of the teeth, alveoli, periosteum and gums; as also dead and loose teeth, and decayed roots, or stumps of teeth, and tartar; all of which will be generally, more or less, observed to accompany the diseases of the maxillæ."

These local causes are, of course, aggravated by all constitutional derangements, whether of an acute or chronic nature—and also by all irregularities of diet and other debilitating causes. The unskilful and severe operations too often performed on the teeth, are not to be overlooked in the chain of causation.

After commenting on various modes which have been employed for destroying the vitality of the teeth, Mr. K. makes the following remark on an operation now in vogue.

"But the operation of breaking or cutting off the crown of painful teeth, which the inventor calls excision, is nothing less than an amputation by violent means, and cannot be adopted from any other cause than a culpable timidity on the part of the patient or the dentist, who are thus led to substitute it for the necessary extraction of the teeth, without even preserving the only useful and essential part, viz. its crown. It unquestionably effects, although not either without pain, or so instantaneously as it is asserted, a destruction of the vitality of the remaining roots or stumps which then become extraneous bodies ; the permanent irritation of which, however, must tend to excite disease and induce mortification not only in adjoining parts, but also in the remaining teeth and gums, not to mention the very great and dangerous irritation produced at the same time upon the whole nervous system.

"Should this be doubted, I beg to refer every medical and surgical reader to a careful examination of the parts which will evidence the fact; for it will be found, that, in a hundred jaws containing roots or stumps without one single exception, the parts contiguous to the roots exhibit some marks of disease or mortification ; unless, indeed, the teeth have been broken after the death of the subjects from which the bones are taken."

Our author observes, that when the expulsion of the roots of teeth is left to the slow efforts of Nature, " a total destruction of the alveoli is the inevitable consequence, and not unfrequently very considerable portions of the bony structure of the jaw will perish through the diseased action." If the tooth be timely removed, not more than the extreme process of the alveoli is generally absorbed.

We must pass over the sections on inflammation and suppuration of the maxilla, fistulous perforations, malignant cancerous affections, &c. and shall merely give a summary of one of the cases detailed in the work.

*Case.* Capt. M. of the East India Company, had laboured under a distressing and complicated disease of the mouth, from an excessive use, or abuse, of mercury, at Calcutta, eleven months previously. On his arrival in England, he went to Mr. Lawrence, who directed him to our author, 26th June, 1826.

"The patient was a tall, well-formed, handsome young man, about twenty-one years of age. According to his own statement his health was originally excellent, and his constitution strong, and only one year previously he was in the possession of a complete set of teeth, they, as well as all their contiguous parts being perfectly sound, regular and beautiful; this was still evident, from the appearance of the remaining parts, which in the morbid and dead state evinced the most striking evidence of their previous perfection.

"All the teeth, although entirely free from caries, or any disease of their bony structure, were now perfectly dead, and only mechanically held in their sockets. The periosteum was also totally destroyed, either by absorption or corrosion. The alveoli were not only dead, but in a state of putrefaction, their upper edges all round the semicircle of the mouth being from an eighth to a quarter of an inch exposed, and exhibiting from their cadaverous appearance a very frightful aspect. The gums were partially destroyed, and the remaining portion of them either gangrenous and sloughing, or in a state of inflammation and suppuration. The disease had already extended to the maxillary bones, and their osseous structure as well as the periosteum of their cavities was more or less under the influence of inflammation, suppuration, and mortification; but more especially the left side of the upper jaw, which was already much increased in size, accompanied with a correspondent swelling of the cheeks. The face was flushed, and the skin had a bloated, erysipelatous appearance, and the patient suffered excessive pain of the whole mouth, the jaw-bones and other parts of the head, as well as of other more remote parts of the system.

"There was a constant flow of viscid ropy discharge from the mouth, like that of great salivation, mixed with greenish matter, and accompanied by a fœtid cadaverous odour, emanating from this fluid and the dead and morbid parts, and so exceedingly offensive as to be almost insupportable to the bystander."

The malady was greatly aggravated by numerous adhesions of the muscles of the jaws to each other, by which almost all power of moving the under jaw was lost. In consequence of this, the teeth were mechanically pressed into their dead sockets, and the absorption and exfoliation of these last much retarded.

In addition to these evils, the almost complete closure of the mouth had prevented the patient from taking any solid food for a long time. He was, therefore, excessively debilitated and nervous. The indications were, to relieve the inflammation of the surviving osseous and soft structures, by promoting exfoliation of the carious sockets and other bones, and more especially by the removal of all the dead teeth. This was a difficult matter as may be readily imagined. All the dead teeth and sockets, however, were removed by Mr. K. and Mr. Lawrence, and, by the middle of August, he was able to set off to visit his friends in the country.

As teeth are equally ornamental and useful in this world, and as we believe that bad teeth produce a number of derangements which are little suspected as to their origin, we recommend the present, and the former work of our author, to the candid consideration of the public.

---

